# Digital Health Literacy and Physical Activity Programme for Improvement of Quality of Life in Caregivers of People with Dementia (CAREFIT): Study Protocol

**DOI:** 10.3390/healthcare13111219

**Published:** 2025-05-22

**Authors:** Patricia Ferrero-Sereno, Patricia Palomo-López, María Mendoza-Muñoz, Patricia Luna-Castaño, Raquel Caballero-De la Calle, Laura Muñoz-Bermejo

**Affiliations:** 1 Social Impact and Innovation in Health (INHealth), University Centre of Merida, University of Extremadura, 06800 Mérida, Spain; 2 Research Group Nurse-In, University Alfonso X el Sabio, Villanueva de la Cañada, Comunidad de, 28691 Madrid, Spain; plunacas@uax.es (P.L.-C.); rdelacab@uax.es (R.C.-D.l.C.); 3 University Centre of Plasencia, University of Extremadura, 10600 Plasencia, Spain; patibiom@unex.es; 4 Research Group on Physical and Heath Literacy and Health-Related Quality of Life (PHYQOL), Faculty of Sports Sciences, University of Extremadura, 10003 Cáceres, Spain; mamendozam@unex.es

**Keywords:** informal caregivers, internet-based intervention, quality of life, occupational stress, anxiety, depression, caregiver burden, social support

## Abstract

**Background/Objectives**: Dementia involves progressive cognitive and functional deterioration that leads to dependence and overload on family caregivers. This overload has a negative impact on the physical, mental, emotional, and occupational health of caregivers, leading to occupational imbalance and problems arising from an inadequate distribution of time devoted to caregiving. This project aims to evaluate the effects of the technology-based CAREFIT programme, structured around physical activity interventions, education, and psychoemotional and social support, on the health-related quality of life and emotional well-being of informal caregivers. **Methods**: The experimental group will develop the intervention programme, which will last 8 weeks and combine educational activities, physical activities, and psychoemotional and social support. Before beginning the intervention, the entire experimental group will receive a training session and educational materials on how to access and use the platform. The CAREFIT platform will consist of two educational sessions and two weekly physical sessions, combined with psychoemotional and social support activities that participants must complete. Initial, final, and follow-up evaluations will be conducted. The HRQoL and psychoemotional health (stress, anxiety, depression, and perceived social support and burden) of caregivers of people with dementia will be the main outcome measures. The effects of the intervention on the study variables will be assessed using a repeated-measures analysis of variance (ANOVA). **Conclusions**: The proposed protocol for the CAREFIT programme represents an innovative and multidisciplinary initiative that leverages a digital platform to promote the well-being of informal caregivers of people with dementia. This approach combines health literacy and strengthened psychoemotional and social support. Through this integration, the goal is to reduce the levels of burden, stress, anxiety, and depression among primary caregivers, while strengthening their self-care capabilities and social support networks.

## 1. Introduction

The increase in the life expectancy of the world’s population and the risk of dementia are growing proportionally. As people age, the likelihood of developing dementia increases, which, in turn, leads to a greater demand for care by older people, especially in Western industrialised countries [1]. Today, an estimated 47 million people worldwide suffer from dementia [2]. The prevalence of dementia increases by approximately 5% for every 5-year increase in age, and is more pronounced in women due to their greater longevity [3].

Dementia is an acquired syndrome characterised by a set of symptoms that result in the persistent impairment of cognitive functions and impaired functional ability in people who do not suffer from impaired consciousness [4]. Dementia, therefore, causes a gradual loss of self-care capacity and increases, over time, the need to receive help from a caregiver to carry out activities of daily living (ADLs). The situation of dependency of the patient with dementia generates a state of overload in the caregiver, who assumes a complex and demanding role. This situation has a significant impact on the caregiver’s health, producing a series of negative consequences [5,6]. In this regard, care for dementia patients falls mainly on family caregivers [7,8]. This role is marked by gender, with women being the main caregivers [9], and also by age, a particularly relevant factor since, in most studies with caregivers, participants are around 65 years of age on average [9,10] and can even reach 77 years of age [11].

The complexity of caring for a patient with dementia generates a significant overload on the caregiver, affecting their physical, mental, and emotional health. The role of the caregiver can generate an overload that negatively affects the occupational performance of informal caregivers, understood as the person’s ability to carry out ADLs (self-care, education/work, play/leisure, social participation, and rest), and instrumental ADLs (those related to caring for others, the home, and the community) [12,13]. One of the consequences or situations of occupational injustice that the informal caregiver may experience is occupational imbalance, which is related to an inadequate distribution of time spent in different occupations. In this context, this involves spending too much time in one area of life and neglecting others, which can lead to various health problems [14].

Therefore, it is especially necessary to design and implement interventions that enable carers to develop strategies and skills to more effectively face the challenge of caregiving, as well as to improve stress management, time management, and access to resources and support services, fostering active ageing and a better quality of life, preparing them to face the physical and mental demands of caregiving and prevent chronic diseases. Furthermore, these interventions should be oriented towards the creation of social and emotional support networks where carers can share experiences, receive advice, and feel more accompanied or supported in their task. Therefore, interventions related to active ageing and quality of life in informal carers of people with dementia are essential to promote their physical, mental, and social well-being and are a necessary investment to ensure the well-being of this population group and contribute to a more sustainable and equitable dementia care system.

Given all of the above, caregiver health literacy can be key to the health of informal caregivers. One of the most recent definitions of health literacy refers to a person’s ability to access, interpret, and apply information and knowledge appropriately in order to preserve and promote their health, taking into account both their personal situation and the health system environment [15], thus including the knowledge, processing, and utilisation of information and the capacity to self-manage it. Numerous studies have shown that there is low health literacy in the adult population [16], as well as in caregivers of people with dementia [17]. This is especially important, as poor health literacy can have a negative impact on the quality of care provided, as caregivers do not have a good understanding of medical concepts or health recommendations and may not apply them correctly. Moreover, it also has an impact on the caregiver themselves, as health literacy has been shown to be related to caregiver burden, health-related quality of life, and time spent on care [18]. In this regard, it is also relevant to consider the factors that influence health literacy, as older age, in particular being over 65 years old, is often considered to be an important factor, but it is a lower educational or socioeconomic status that is generally associated with lower health literacy [19].

Specifically, in relation to physical activity, previous studies have shown that informal carers of people with dementia are more likely to report substantial physical difficulties in providing care to their beneficiaries compared to carers of people without dementia, and that when care is provided to their spouses, they are significantly more likely (41%) than other co-spousal carers of a similar age to become increasingly frail during the time between becoming carers and the death of their spouse [20,21].

Thus, physical activity could play a crucial role, as the benefits of physical activity for the adult population, both physically and mentally, are well known. However, until a few years ago, there was no evidence on the specific benefits that physical activity could have specifically on informal caregivers of people with dementia, but more and more recent studies have looked at how physical activity affects this population. In 2023, da Silva-Sauer et al. [22] found that there was a significant association between physical activity restriction, increased somatic symptoms, and increased caregiving burden among informal caregivers, particularly with regard to cardiovascular complaints. The researchers emphasised the importance of addressing caregivers’ sedentary lifestyles and promoting exercise to improve their health and quality of life. Consequently, physical activity interventions could have a beneficial effect on caregivers’ psychosocial aspects and physical activity levels, with caregivers themselves stating that physical activity can be an important element in their emotional health, physical well-being, and social connectedness [23].

The inclusion of technologies as a means of developing PA and health interventions could be very effective, as a growing body of scientific evidence supports the effectiveness of technology-based interventions for improving health and well-being [24,25,26], being generally effective in improving depressive symptoms, perceived stress, anxiety, and self-efficacy in family caregivers of people with dementia and having potential benefits for those receiving care [24]. Digital interventions provide numerous advantages, such as (a) high accessibility, being able to be used at any time and any place; (b) flexibility, adapting to the individual needs and preferences of each caregiver; (c) interactivity, offering interactive content, such as videos, games, and quizzes, to facilitate learning; and (d) personalisation, adapting to the physical levels and emotional state of each caregiver, as well as recommending personalised resources based on the caregiver’s needs or time.

To the best of our knowledge, there have been studies that intervened through psychoeducational [27,28] or cognitive–behavioural [29,30] programmes for caregivers of people with dementia or publications on didactic exercise interventions in people with dementia and their caregivers [31,32], as well as some educational, psychoemotional, and physical interventions whose medium of application was the internet [24]. In addition, there is evidence that programmes including educational modules, forums and real-time support sessions [33], and eHealth psychoeducational programmes aimed at increasing knowledge of neurocognitive disorders, their management at home, and the presence of national support services [34] have significantly reduced the level of behavioural symptoms in patients with dementia, increased empowerment, and eased the burden on caregivers, thus improving their caregiving skills. There are even interventions that have evaluated the effectiveness of an online psychoeducational programme combined with an immersive virtual reality (VR) experience in reducing caregivers’ perceived stress by acting on the cognitive and affective components of empathy [35]. However, there is no study that has used technological means to carry out an interdisciplinary programme with PA interventions together with health literacy for informal caregivers of people with dementia. Therefore, the aim of this project is to determine the effect of an 8-week intervention programme based on the CAREFIT (Caregivers’ Active Resilience & Exercise for Fitness and Well-being) programme on the HRQoL and psychoemotional health (stress, anxiety, depression, and perceived social support and caregiver burden) of caregivers of people with dementia.

In this sense, the hypothesis of the study protocol is that the implementation of an 8-week intervention programme based on the CAREFIT model will improve health-related quality of life (HRQoL) and psychoemotional health indicators (decreased stress, anxiety, depression, and overload, as well as increased perceived social support) in caregivers of people with dementia, compared to those who do not participate in the programme.

## 2. Materials and Methods

### 2.1. Design and Description of the Programme and Its Contents

Following other studies targeting caregivers of people with dementia, a telephone- and Internet-based intervention is proposed [36]. In line with previous studies [34,35], the CAREFIT programme will be developed using WordPress, a widely used content management system (CMS) running PHP and MySQL. According to W3Techs [37], more than 28% of the top ten million websites use this platform. Thanks to its open-source nature and large community of developers, WordPress offers solid online support and a great capacity for expansion through plugins.

The main menu will be divided into sections for physical, educational, psychoemotional health, and social support activities.

The physical activities section will include sessions with a variety of exercises, ranging from stretching to exercises aimed at improving strength, balance, or endurance. All sessions will feature videos guided by an avatar, who will time each exercise and session. This avatar will narrate the objective and key points for the execution of each exercise before it starts, as well as the beginning and end of the exercise. For each exercise, there will be three levels (basic, intermediate, and advanced) that can be selected by the participant prior to execution, thus adapting to their level.

The educational section will provide training on the benefits of healthy living habits, resources on self-care and family care, as well as on how to incorporate these into ADLs or daily life, based on multimedia presentations and interactive or animated infographics or videos.

The psychoemotional support section will offer resources to prevent caregiver burden, stress, anxiety, and depression.

The social support section will offer resources and tips for caregivers, such as information on support networks, dependency care services, and strategies for managing stress, overload, and self-care.

The planning of the interventions is presented in Table 1.

In addition, to measure adherence to the intervention, an electronic tracking system will be used to monitor participants’ attendance rates at sessions, the completion of tasks or activities, and the percentage of participants who complete the programme compared to those who started it. Throughout the intervention, the research team will be in frequent contact with the participants through phone calls and messages in order to provide support and motivation, thus encouraging their involvement in the process. In addition, participants will receive a monthly report summarising their progress and achievements.

### 2.2. Randomised Controlled Trial

#### 2.2.1. Desing

A Randomised Controlled Trial (RCT) will be conducted, designed following the Consolidated Standards of Reporting Trials (CONSORT) [38] for the conduct of this type of design. It will comprise an 8-week intervention phase, including an assessment at baseline, an assessment upon completion, and a follow-up assessment one month after the end of the programme. Random sampling will be used. Participating caregivers will be randomly assigned to either the intervention (experimental) or the “usual care” (control) group.

#### 2.2.2. Ethics

Ethical approval for this study was obtained from the Bioethics and Biosafety Committee of the University of Extremadura, with approval number (145/2024). This ensures that the research adheres to the highest ethical standards, prioritising the welfare and rights of all participants involved. In addition to institutional approval, the study protocol has been registered with OFS (osf.io/93trc). This registration serves to increase transparency and accountability in the research process by allowing public access to the details and methodology of the study. By obtaining both ethics approval and trial registration, the study aims to uphold the principles of ethical conduct in research, ensuring that participant safety and informed consent are prioritised throughout the duration of the trial. This commitment to ethical standards is essential to foster trust and integrity in the research community.

#### 2.2.3. Sample Calculation

The calculation of the number of participants needed for this study was based on the expected change in quality of life as measured by the EQ-5D-5L questionnaire. Since, to our knowledge, there are no specific data on the minimum clinically relevant change in informal caregivers using this instrument, a minimum difference of 0.07 units was taken as a reference value [39]. Considering a significance level (alpha) of 0.05 and a statistical power greater than 0.8 in a two-sided test, it was determined that 25 participants in the control group and 25 in the experimental group are needed to identify a difference equal to or greater than 0.07 units as significant. A common standard deviation of 0.12 [40] and a correlation coefficient of 0.8 between the initial and final measurements were assumed. In addition, an estimated dropout rate of 25% was calculated. The calculation was performed using version 7.12 of the GRANMO to determine the sample size. (https://www.datarus.eu/aplicaciones/granmo/ (accessed on 10 January 2025)).

#### 2.2.4. Participants

Informal carers of people with dementia will be recruited from specific associations and social and healthcare centres by the means of non-probabilistic convenience sampling. For this purpose, project information sessions will be held at the different associations and socio-health centres in the region, and leaflets will be distributed for recruitment. The research team will identify potential participants and assess them for eligibility according to the inclusion criteria specified below.

Participants have to be a primary informal caregiver of a person with a diagnosis of dementia, dedicating more than 20 h per week to this task for a minimum of three months, and be willing to continue providing care for the next year. They cannot have taken part in or received any type of psychoeducational or cognitive–behavioural therapy-based intervention in the three months prior to the start of the study. They must complete and sign both the informed consent and the acceptance document to participate in the study. They must have a mobile device or, failing that, a tablet or computer.

#### 2.2.5. Intervention

**Figure 1:**
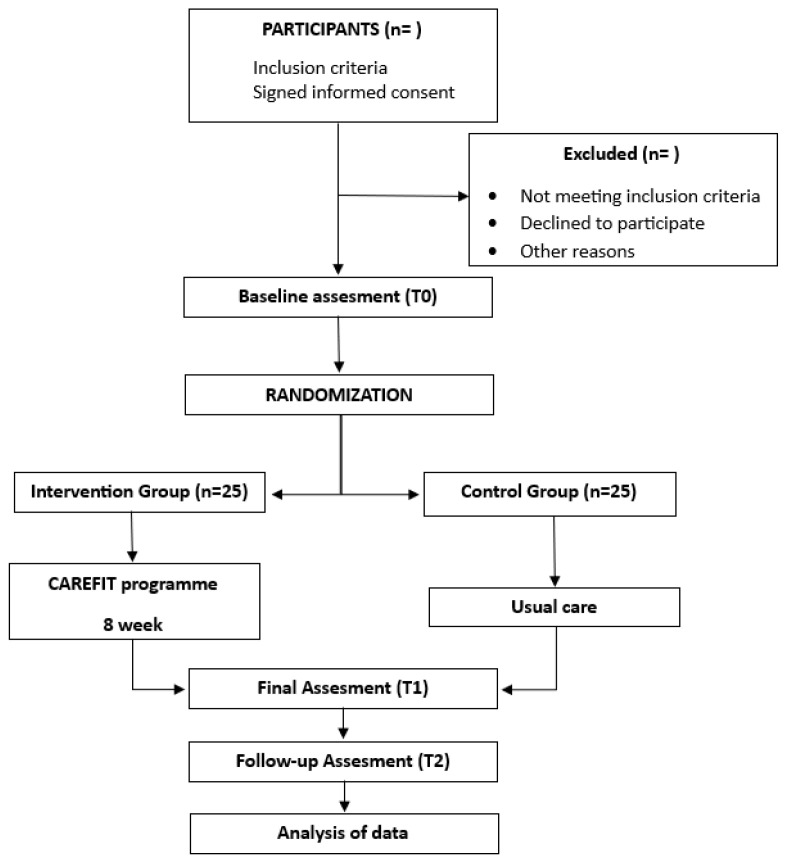
Flow chart for the RCT.

After completing the initial assessments, all participants will be randomly allocated between the experimental intervention group and the control group. To assign participants, a random sequence will be generated in a 1:1 ratio, which will assign participants to either the experimental or control group. This will be performed using Research Randomizer software (version 4.0, Geoffrey C. Urbaniak y Scott Plous, Middletown, CT, EE. UU.; http://www.randomizer.org (accessed on 16 June 2024)) [41]. This allocation will be performed by a member of the research team who has no clinical involvement in the study. The allocation information will be stored confidentially in a password-protected file. Although participants will know which group they have been assigned to, outcome assessors and data analysts will have no knowledge of their assignment. The experimental group will develop the intervention programme, which will last 8 weeks and will combine educational activities, physical activities, and psychoemotional and social support. The programme will be designed to be flexible and adaptable to the individual needs of the caregivers. Before starting the intervention, the entire experimental group will receive a training session and training material on how to access and use the platform. In addition, once a week, a member of the research team will have a telephone or video call with each participant to solve doubts or technical problems and to follow up in order to adjust the intervention to the individual needs or personal situations of each caregiver. The CAREFIT platform will contain 2 educational sessions and 2 physical sessions per week combined with psychoemotional and social support activities to be completed by the participants. The control group will carry out their usual daily activities. The flow chart for the RCT is shown in Figure 1.

#### 2.2.6. Measurements and Instrument

The measures to be carried out will consist of a questionnaire booklet that will include the assessment of socio-demographic data (age, sex, level of education, income, occupation, marital status, life habits, and pathologies), HRQoL, caregiver burden, occupational balance, satisfaction with daily occupations, depressive symptoms, anxiety, stress, and perceived social support.

The following instruments will be used for this purpose:

(1) Quality of life
  - EQ-5D-5L. This questionnaire measures the health status of participants and is divided into the following three assessments: (1) the assessment of several dimensions of health (mobility, self-care, activities of daily living, pain/discomfort, and anxiety/depression) using a descriptive Likert-type system from 1 to 5 points (no problems to problems/external impossibility); (2) the assessment of health status using a visual analogue scale; and (3) the assessment of social value using an index generated from the health states obtained in the first level [42]. It is a valid and reliable questionnaire both in the Spanish population [43] and in other populations [44] and in caregivers of people with other pathologies, with a validity of 0.987 [45].
(2) Stress
  - Perceived Stress Scale (PSS) [46]. This scale will measure perceived stress and consists of 14 questions that measure the degree to which situations in a person’s life are rated as stressful. The questions are formulated in terms of feelings and thoughts over the last month, and participants are asked to rate the frequency with which they have experienced them on a 5-point Likert scale. The score will be used to assess the participants’ level of stress. This scale showed a good reliability in the Spanish population, with a Cronbach’s alpha 0.82 [47].
(3) Anxiety and depression
  - General Health Questionnaire (GHQ-12) [48]. This scale will be used for the assessment of depression and anxiety. It is composed of 12 items (Yes/No). The cut-off point for the anxiety subscale is 4 or more points and 2 or more points for the depression subscale. Higher point values indicate a more severe problem, with 6 being the highest possible value for each subscale. The internal consistency analysis in Spanish population yielded an alpha coefficient of 0.78 [49].
  - Geriatric Depression Scale (GDS). This questionnaire will measure depression and consists of 15 questions about how the participant has been feeling in the last 14 days, with the answers limited to ‘yes’ or ‘no’. The internal consistency analysis yielded an alpha coefficient of 0.87 [50].
(4) Caregiver burden
  - Zarit Burden Inventory (ZBI) (Spanish version). This 22-item questionnaire quantifies caregiver strain. This scale consists of 22 items in a Likert scale format, using a scale from one (never) to five (almost always). The recommended cut-off points are as follows: <46 indicating no caregiver burden; 46 to 56 items indicating mild caregiver burden, and >56 items indicating severe caregiver burden. Its internal consistency is high, with a Cronbach’s alpha coefficient of 0.91 [51].
(5) Occupational balance
  - Occupational Balance Questionnaire (OBQ-E). This questionnaire assesses participants’ satisfaction with their occupations by the means of 13 items, answered on a Likert scale from zero ‘strongly disagree’ to five ‘strongly agree’. The internal consistency obtained a value of Cronbach’s alpha of 0.948 [52].
(6) Perceived social support
  - Functional Social Support Questionnaire DukeUNC-11 [53]. This instrument consists of 11 items that are answered on a Likert scale from 1 to 5, where 1 represents ‘much less than I want’ and 5 ‘as much as I want’, where the questionnaire score ranges from 11 to 55 points. A score of 32 or above indicates standard social support, while a score below 32 suggests low perceived social support. In the Spanish population, the internal consistency of the questionnaire was found to be high, with a consistency coefficient of 0.90 [53].

Assessments will be conducted before the start of the intervention (baseline), at the end of the intervention (8 weeks), and one month after the end of the intervention (12 weeks) (Table 2).

#### 2.2.7. Statistical Analysis

The data will be analysed with the computer programme IBM Social Sciences (SPSS, version 25.0, Armonk, NY, USA). The normality and homogeneity of the data will be examined by Levene and Kolmogorov–Smirnov tests, respectively. The baseline and socio-demographic characteristics of the study participants will be presented for categorical variables as frequencies and percentages and for continuous variables as means and standard deviations.

The effects of the intervention on the study variables, HRQoL, physical condition, and psychoemotional state (stress, anxiety, depression, caregiver strain, and perceived social support), will be assessed by repeated-measures analysis of variance (ANOVA). The results of the study will incorporate the effect size, together with its corresponding 95% confidence interval, as well as the statistical significance for each measure analysed, considering both the evolution over time and the interaction effects between group and time. If the data do not follow a normal distribution, the Friedman test will be applied. Statistical significance will be established at the conventional level of *p* < 0.05.

In the case of missing data, the Intention-To-Treat (ITT) strategy will be used to maintain the original randomisation and reduce the risk of bias by analysing all participants according to the group to which they were assigned, regardless of their level of adherence.

## 3. Discussion

This research protocol is innovative in its approach to physical activity and health promotion for informal caregivers of people with dementia. The expected findings will show how physical activity, access to training courses, and psychoemotional and social support have a positive impact on the quality of life (HRQoL) and psychoemotional health of informal caregivers [2].

Firstly, it has been identified that the global increase in life expectancy and the prevalence of dementia generate an increase in the demand for care [3]. This situation translates into increased strain on caregivers, mostly elderly women, who experience physical and emotional overload [9,10]. Health literacy is, therefore, of particular relevance, since, as pointed out by Liu, Wang, Liu, Jiang, Wang, Chen, Ju, and Zhang [15], this enables carers to obtain, process, and apply information to maintain and improve their health in a way that is tailored to their particular context.

Currently, the literature shows insufficient health literacy among caregivers [17], a fact that influences both the quality of care provided and the caregiver’s well-being [18]. In particular, it has been observed that caregivers with low health literacy may have higher levels of caregiver strain, a lower quality of life, and even spend more time on caregiving due to a lack of effective coping strategies [19]. In this sense, the CAREFIT programme addresses this knowledge gap by offering, through the platform, training and support resources to promote self-care.

The benefits of physical activity in the adult population improve both physical fitness and mental health [20,21]. The case of informal caregivers of people with dementia is of particular importance, given that many of them start with a poor physical condition [20]. According to da Silva-Sauer, Garcia, Fonsêca, and Fernández-Calvo [22], the restriction of physical activity is associated with increased somatic complaints and an increased caregiving burden. Farina, Williams, Clarke, Hughes, Thomas, Lowry, and Banerjee [23] emphasise the importance that caregivers attach to physical activity for their own emotional and social well-being. Under this premise, the inclusion of adapted exercise and active living patterns in CAREFIT has the potential to improve cardiovascular health and general fitness, as well as being a protective factor against stress and depression [24,25,26].

Given the multicomponent nature of the CAREFIT intervention programme, focusing on physical activity, resilience, and psychoemotional well-being, it is anticipated that its effectiveness could vary according to the specific socio-demographic characteristics of participating caregivers, such as age, gender, and educational level.

In this sense, younger caregivers may show a greater capacity for physical adaptation to the active components of the programme, which would favour significant improvements in their general physical state and, therefore, in their perception of health-related quality of life (HRQoL). However, older caregivers may benefit more from the emotional and social component, as they tend to experience a greater degree of isolation and sustained emotional burden. In these cases, a focus on resilience and perceived social support may be particularly effective in mitigating symptoms of anxiety, depression, and caregiver burden [54]. On the other hand, studies indicate that women are more likely to assume the role of primary caregivers and, as a result, report higher levels of stress and strain. In this sense, the programme is expected to have a particularly positive impact on female caregivers by providing them with coping tools and opportunities for self-care [55]. In contrast, male caregivers, although less represented in this role, may experience benefits in terms of emotional connection and stress management, aspects that are often less developed in this group due to traditional social norms [56]. Finally, level of education may influence understanding and adherence to the programme. Caregivers with a higher level of education may show a better assimilation of the psychoeducational contents and a greater willingness to implement the proposed changes in their daily routine [57]. However, less educated carers are not exempt from the benefits; for this group, accessibility of language, clarity of instructions, and close accompaniment will be key factors in encouraging active and sustained participation [58].

In this sense, the adoption of digital resources not only facilitates the accessibility of information, but also enables personalisation and the continuous monitoring of adherence to the programme, overcoming geographical or logistical barriers [27,28]. In addition, the integration of psychoeducational and emotional support content, such as in the work of Cristancho-Lacroix, Wrobel, Cantegreil-Kallen, Dub, Rouquette, and Rigaud [28], offers a closer accompaniment to the caregiver, favouring the reduction in caregiver burden and the improvement of self-confidence in the management of care situations.

Furthermore, the multidisciplinary approach, encompassing educational support, physical activity, psychoemotional support, and social support, is in line with evidence indicating that interventions in multiple areas tend to report better outcomes in terms of adherence and effectiveness [29,32]. For example, Lamotte, Shah, Lazarov, and Corcos [32] evidence that physical exercise programmes, coupled with an educational component, improve quality of life and reduce caregiver burden. This comprehensive approach can be instrumental in promoting sustainable changes in caregivers’ lifestyles and maintenance of their own health. This is why the present protocol can bring health literacy benefits, improved quality of life, improved physical condition, and emotional support using a technological application, which can be adapted to each caregivers’ rhythm of life. In this sense, it has been observed that on-demand interventions, where the caregiver can perform activities without a set frequency, have had better results [59]. In short, the use of technological solutions has proven to be an effective solution to reduce caregiver burden, improve quality of life, and avoid the negative physical and psychological consequences of caring for a dependent person [60,61]. However, in order to maintain the long-term effects of the CAREFIT programme on caregivers of people with dementia, it is essential to incorporate follow-up, reinforcement, and sustainability strategies that ensure the integration of the learned habits into daily life. Finally, regarding the strengths of the study, this study could play a key role in reducing the social isolation of carers, promoting their social inclusion and the creation of support networks. Ultimately, the CAREFIT platform is designed to respond to the specific needs of carers, offering tailored and relevant resources that can make a significant difference in their daily lives. Facilitating active ageing and improving the quality of care provided are central objectives of this initiative. In addition, one of the greatest strengths is the importance of the transferability of the results of this project. If the intervention proves effective, its applicability could be extended not only to caregivers of people with dementia, but also to other informal care settings for chronically ill patients. Furthermore, the incorporation of the platform into public health services or institutional programmes could facilitate a standardised and low-cost intervention, with positive repercussions in reducing the high social and healthcare costs derived from the overburden of caregivers [6,10]. This would favour the implementation of more efficient support policies, aimed at both the promotion of the carer’s health and the improvement of the care provided to the patient.

However, it is important to consider some possible limitations to the implementation of the platform. These include the need for financial and technological resources for maintenance and upgrading, as well as training and support for health and education professionals responsible for implementing the programme in real-world settings [62]. In addition, a limitation to consider is the non-probabilistic convenience sampling and the risk of digital exclusion for some caregivers. Some limitations that could affect technological adherence in this group would be low digital literacy, which could lead to insecurity, frustration, and dropout from the programme. Also, the lack of access to devices or internet could limit participation, especially in rural areas or those with a lower socio-economic status. However, given that it is a low-cost healthcare technology that can be easily standardised at different levels of difficulty, its transfer to and implementation in both the public and private sectors could be feasible and sustainable in the long term.

## 4. Conclusions

The proposed protocol for the CAREFIT programme, based on a digital platform, offers an innovative and multidisciplinary approach using technology to improve the quality of life of informal caregivers of people with dementia. By integrating health and physical activity literacy with psychoemotional and social support, it is expected to reduce overload, stress, anxiety, and depression in informal primary caregivers, as well as strengthen their support networks and self-care skills.

Given that the planned technology-assisted interventions meet the requirements of flexibility and accessibility, it will facilitate the acquisition of healthy lifestyle habits and favour an improvement in mental health. If the programme proves to be effective, its results could be applied more widely in the social health field, being incorporated into public or private care policies and plans, in order to support the well-being of both caregivers and dementia patients themselves.

## Figures and Tables

**Table 1:** Session characterisation.

Section	Modality	Module
Physical activities section	Virtual	Ranging from stretching
		Strength, balance, or endurance
Educational section	Virtual	Healthy lifestyle habits
		Self-care resources
		Family care resources
Psychoemotional support section		Overload
	Virtual	Stress
		Anxiety
		Depression
Social support section	Virtual	Information on support networks Care services for dependent persons

**Table 2:** Assessments scheduled for both experimental and control groups.

Assessment	Baseline	Final	Follow-Up
Socio-demographic data	X		
Quality of Life	X	X	X
Stress	X	X	X
Anxiety	X	X	X
Depression	X	X	X
Caregiver burden	X	X	X
Occupational balance	X	X	X
Perceived social support	X	X	X

## Data Availability

Not applicable.
